# A localization strategy combined with transfer learning for image annotation

**DOI:** 10.1371/journal.pone.0260758

**Published:** 2021-12-08

**Authors:** Zhiqiang Chen, Leelavathi Rajamanickam, Jianfang Cao, Aidi Zhao, Xiaohui Hu

**Affiliations:** 1 Information Technology, SEGi University, Kota Damansara, Petaling Jaya, Selangor, Malaysia; 2 Department of Computer Science and Technology, Xinzhou Teachers University, Xinzhou, China; 3 School of Computer Science and Technology, Taiyuan University of Science and Technology, Taiyuan, China; National University of Sciences and Technology, PAKISTAN

## Abstract

This study aims to solve the overfitting problem caused by insufficient labeled images in the automatic image annotation field. We propose a transfer learning model called CNN-2L that incorporates the label localization strategy described in this study. The model consists of an InceptionV3 network pretrained on the ImageNet dataset and a label localization algorithm. First, the pretrained InceptionV3 network extracts features from the target dataset that are used to train a specific classifier and fine-tune the entire network to obtain an optimal model. Then, the obtained model is used to derive the probabilities of the predicted labels. For this purpose, we introduce a squeeze and excitation (SE) module into the network architecture that augments the useful feature information, inhibits useless feature information, and conducts feature reweighting. Next, we perform label localization to obtain the label probabilities and determine the final label set for each image. During this process, the number of labels must be determined. The optimal K value is obtained experimentally and used to determine the number of predicted labels, thereby solving the empty label set problem that occurs when the predicted label values of images are below a fixed threshold. Experiments on the Corel5k multilabel image dataset verify that CNN-2L improves the labeling precision by 18% and 15% compared with the traditional multiple-Bernoulli relevance model (MBRM) and joint equal contribution (JEC) algorithms, respectively, and it improves the recall by 6% compared with JEC. Additionally, it improves the precision by 20% and 11% compared with the deep learning methods Weight-KNN and adaptive hypergraph learning (AHL), respectively. Although CNN-2L fails to improve the recall compared with the semantic extension model (SEM), it improves the comprehensive index of the *F1* value by 1%. The experimental results reveal that the proposed transfer learning model based on a label localization strategy is effective for automatic image annotation and substantially boosts the multilabel image annotation performance.

## Introduction

The development of multimedia technology has increased the amounts of all types of multimedia data. As the main representative of multimedia data, images have been the primary focus of many studies. At present, methods of classifying single objects in images have become highly sophisticated. However, in real life, images often include multiple objects; thus, relying on only a single keyword to represent image semantics is often insufficient. The multilabel image annotation field has emerged to solve this problem. By assigning multiple labels to an image, the labels more accurately capture the true image semantics and better match the real world.

In recent years, traditional machine learning methods have been widely applied in automatic image annotation models; for example, ML-KNN [1], developed from K-nearest neighbors (KNN), is used in the multilabel annotation model. This method first finds the K neighbors closest to the sample, counts the labels of those K neighbors, and then selects the label set for the sample based on the maximum a posteriori probability (MAP) score. However, this method is relatively complex. When the samples are imbalanced, KNN is poor at predicting rare categories. Xiang et al. [2] studied a semantic context modeling and learning approach for automatic image annotation based on multiple Markov random fields (MMRFs). This approach performs annotation by estimating the joint probability distribution of cooccurrences of semantic concepts and images. However, although this joint distribution can provide more information, it requires additional samples and computation, which may result in a waste of computing resources during classification. Shi et al. [3] proposed a feature selection framework with enhanced sparsity that uses the *l*_2,1/2_-matrix norm with shared subspace learning to select the sparsest and most discriminative features while taking the correlations between different features into account. However, selecting only sparse features may cause some relevant information to be removed, thereby undermining the image annotation effect. Thus, based on an improved support vector machine (SVM) method [4], they established multiple classifiers and created an SVM multilabel classification model that uses distance as a discriminative index. This method generates good results on small training sets. However, it results in excessive machine memory and computational time when applied to large datasets. Li et al. [5] proposed an improved image annotation method based on fuzzy C-means (FCM) clustering that improved the distance measure and replaced the previous distance measure by the differences in the distances between similar and heterogeneous samples. However, this method does not guarantee that the optimal problem solution will be found and may converge to a local optimum. Yuan et al. [6] proposed the multiple kernel learning with group sparsity (MKLGS) method, which selects groups of discriminative features for image annotation based on the relative importance of different high-level semantic features. The weakness of this method is that it does not support selection between and within feature groups at the same time. Although the above methods have achieved encouraging results in the image annotation field, traditional machine learning methods still suffer from numerous shortcomings in extracting image features: 1) manual feature extraction is time- and labor-intensive, making it relatively expensive; 2) manual feature selection inevitably leads to information loss, which results in reduced precision and recall in experimental results [7].

Deep learning techniques have achieved impressive results in the field of computer vision; among these, various convolutional neural network (CNN) models have demonstrated outstanding performances in image classification. In recent years, CNN classification performances have surpassed even those of humans in terms of precision. For example, GoogLeNet [8], which was proposed in 2014, was the first to surpass manual recognition and classification on the ImageNet dataset. Li et al. [9], Szegedy et al. [10] and He et al. [11] proposed a multilabel automatic image annotation method based on an improved AlexNet [12] model. Based on the loss function of a single-label CNN, Li et al. [9] designed a new multilabel loss function based on softmax regression. Ke et al. [13] proposed an automatic image annotation method that combined semantic neighbors and deep features and used a CNN to extract features and construct neighborhood image sets that were similar in terms of both vision and semantics; then, they used the distances between sets to sort the labels before annotation. Salma et al. [14] proposed HierarchyNet, which is also based on a CNN and is similar to the branch convolutional neural network (B-CNN) proposed by Zhu & Bain [15], which introduced a nonoverlapping hierarchical coarse-to-fine tree to improve the target classification. This method uses multiple branch classifiers so that classification corresponds to different layers in the tree, arranging the prediction results in coarse-to-fine order. Chen et al. [16] proposed a dense residual three-dimensional convolutional neural network (DR3D-CNN). Hyperspectral images use three-dimensional convolution in place of the traditional two-dimensional convolution to effectively extract spectral features. The DR3D-CNN model further refines the initial network classification using multilabel conditional random field optimization. Markatopoulou et al. [17] proposed an end-to-end deep convolutional neural network (DCNN) architecture in which a CNN was used as a feature generator, and an SVM classifier was trained on the DCNN-generated features. Xu et al. [18] proposed an atrous convolutional feature network (ACFN) composed of a cascaded atrous convolution module and a pyramidal atrous convolution module. This network improved the target attention graph by enhancing the contextual representation ability of the image classification network. In addition, they proposed an attentive fusion strategy that adaptively fused multiscale features. Although deep learning has a remarkable performance advantage for image classification, in real life, the scales of existing multilabel image datasets are too small, which easily results in overfitting during the deep learning process, making it difficult to take full advantage of the capabilities of deep learning networks.

To address problems such as information loss caused by using manual features in traditional machine learning, insufficient datasets in deep learning, and empty prediction label sets caused by a fixed threshold, we propose a transfer learning model based on a label localization strategy. First, we construct a CNN for multilabel labeling based on transfer learning. Using a network weight file trained on large datasets and fine-tuned on smaller target datasets, we extract the feature information from the target datasets, which prevents both the feature loss caused by setting features manually and the overfitting caused by datasets that are too small in deep learning. Second, we introduce the squeeze and excitation (SE) module [19] into the network architecture to achieve feature reweighting through a three-step operation (namely, squeeze, excitation and reweighting) that further improves the network performance. Third, the model predicts all the labels to obtain the prediction probability for all the labels of the current image. Finally, the label set of the current image is determined using a label localization strategy, which solves the empty label set problem caused by a fixed threshold.

The main innovations of this study are summarized as follows:

1. We introduce transfer learning to solve the overfitting problem caused by applying insufficient datasets to the deep learning process. We use a weight file obtained by training an InceptionV3 network on the large ImageNet dataset to extract the features of target datasets, and we adjust the model parameters via the target datasets. Then, we use the binary cross-entropy loss function in place of the previous loss function to adapt the model to the multilabel annotation task and solve the overfitting problem.
2. We introduce the SE module to inhibit useless features and enhance the effective features. In this study, we assign different weights to different features extracted by the convolutional layers using three-step operations (i.e., squeeze, excitation and reweighting) to achieve feature reweighting and further improve network performance.
3. When using a fixed-threshold method, we propose a label localization strategy to avoid empty label sets when the prediction probability of some images is below the fixed threshold. The label localization strategy locates the label probability predicted by the model. By determining the label probability prior to the K value, we can locate and determine the label set for each image. This strategy solves the problem of using a fixed threshold and boosts the annotation accuracy.

## Methods

### Transfer learning model based on the label localization strategy

**Fig 1:**
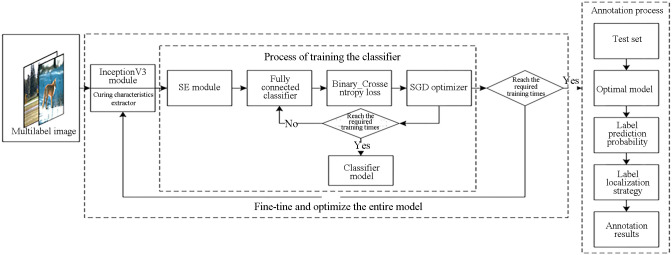
Framework of the multilabel classification algorithm.

The algorithm framework of the transfer learning model based on the label localization strategy is shown in Fig 1. The main steps in this model include training the input layer, feature extraction, classification, fine-tuning, and optimizing both the entire model and the annotation stages. The multilabel images input to the input layer are RGB images uniformly resized to 299 × 299. We adopt an InceptionV3 [20] model pretrained on ImageNet as the feature extractor for multilabel images. During classifier training, we first establish a classifier suitable for the dataset and add a fully connected layer (FCL) with 260 neurons to replace the original FCL. Then, the features saved in the feature extraction stage are used to train the classifier and optimize its weight parameters. During the fine-tuning and optimization stage for the entire model, the weights of the highest convolutional layers of the InceptionV3 network are adjusted to make the weight parameters of the model more suitable for the target dataset.

To explore the relationships between feature channels and further improve the model’s expressive ability, we introduce the SE module, which uses a “feature reweighting” strategy to enhance the useful feature information while suppressing features that are less relevant to the current task. Simultaneously, to make the network model adapt to the multilabel classification task, we use the binary cross-entropy loss function to measure the difference between the predicted value output and the real value. We replace the softmax function of the last layer with a sigmoid activation function to estimate the relevant a posteriori probability of each label. The sigmoid activation function is expressed by Eq (1):

$$\text{Sigmoid}\;\left(x\right)=\frac{1}{1+exp\left(-x\right)},$$ (1)

where *x* represents the activation values of the neurons in the hidden layer prior to the transformation. We adopt stochastic gradient descent (SGD) to optimize the model. To improve the convergence speed during training, the learning rate is set to a large initial value (i.e., 0.1) and then automatically reduced periodically. When classifier training reaches a predetermined number of iterations, the weight file of the classifier is saved, at which point classifier training is complete. The fine-tuning and optimization step for the entire model uses a small learning rate, i.e., 0.0001, to achieve optimal performance.

### Improvements

#### Transfer learning

Transfer learning is the application of knowledge or patterns learned from a certain field or task to a different but related field or problem. Transfer learning can be defined as follows [21]: given a source domain (DS), a source task (TS), a target domain (DT) and a target task (TT), knowledge learned from the DS and TS is utilized to help improve the learning of the DT and performance of the TT.

Deep learning requires many training samples. However, there are few datasets containing labeled multilabel images. Given this circumstance, transfer learning uses a model pretrained on a large dataset and then adjusts the models by quickly learning a target domain dataset to complete the target task [22]. First, a network model M1 is pretrained on the DS and TT of a large dataset. Because the source domain dataset is large, the deep learning model can learn its characteristics well. Then, a new model (M2) is obtained by fine-tuning the M1 model on the DT and TT, resulting in an adjusted M2 model that is more suitable for the DT and TT.

In image processing, the underlying features of images are basically similar, such as color features [23], shape features [24], and texture features [25]. Therefore, a network model pretrained on large datasets can be used as the feature extractor. In this study, we use an InceptionV3 model pretrained on ImageNet as the image feature extractor. The InceptionV3 model introduces the idea of “decomposition”, and it decomposes the larger convolution kernel into two smaller convolution kernels. For example, a 7 × 7 convolution is split into a 1 × 7 convolution and a 7 × 1 convolution. On the one hand, this split reduces the number of network parameters, while on the other hand, it increases the expressive ability of the model. The resulting asymmetric convolution structure after splitting increases feature diversity.

#### SE module

**Fig 2:**
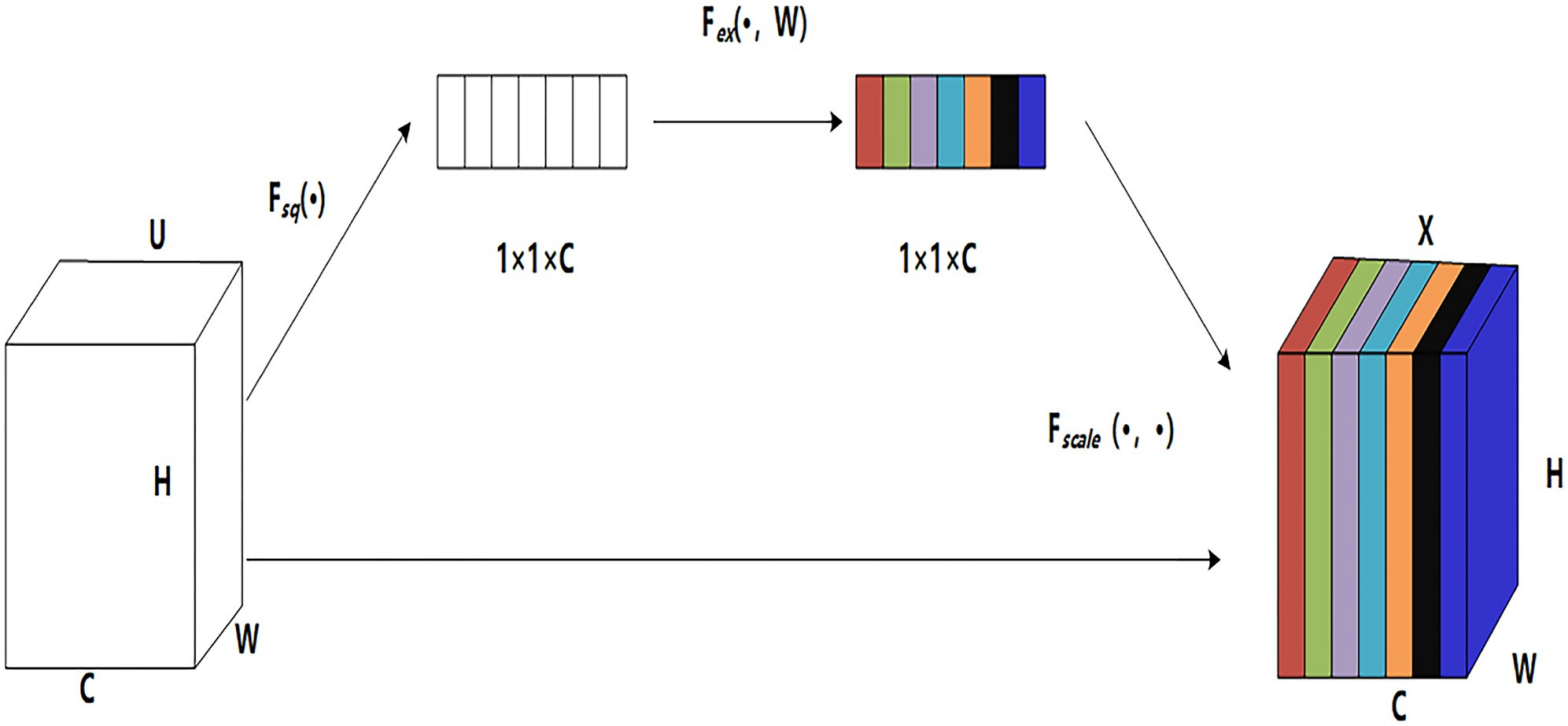
SE module.

To further improve network performance, we focus on the relationships between the feature channels and introduce the SE module into the model to improve the expressive ability of the network model by accurately modeling the interactions among the various feature channels of the convolution layers. The importance of each feature channel is automatically acquired during the learning process. Based on this importance value, the useful features are enhanced, while the features that are less relevant to the current task are suppressed. As shown in Fig 2, the SE module includes three main operations: squeeze, excitation, and reweighting.

In Fig 2, U represents the output of each convolution layer in the backbone network, and X is the output of the feature map after weight fusion. H and W are the height and width dimensions of the feature map, respectively, and C represents the number of channels, that is, the number of feature maps. F_*sq*_(·), F_*ex*_(·) and F_*scale*_(·) represent the three operations, namely, squeeze, excitation and reweighting.

The squeeze operation compresses the spatial feature dimensions and compresses the global spatial information into a channel descriptor using global average pooling. Each two-dimensional feature channel becomes a real number. At this time, the C-th feature map of U is calculated as shown in Eq (2):

$$z_{c}=F_{sq}\left(u_{c}\right)=\frac{1}{H\times W}\sum_{i=1}^{H}\sum_{j=1}^{W}u_{c}\left(i,j\right).$$ (2)

The excitation generates the weights for each feature channel by means of learnable parameters to comprehensively obtain the dependencies between channels. The operation is composed of two FCLs and a sigmoid activation function. The first FCL reduces the feature dimension and the parameters and then adds a ReLU activation layer to increase the nonlinearity. Then, a second FCL is added to restore the dimension. Finally, the input is mapped to the 0~1 interval using the sigmoid function. The excitation calculation is shown in Eq (3):

$$s=F_{ex}\left(z,W\right)=\sigma \left(g\left(z,W\right)\right)=\sigma \left(W_{2}\delta \left(W_{1}z\right)\right)$$ (3)

The dimension of *s* is 1×1×C, and it represents the weights of the C feature maps in U. *W*_1_ and *W*_2_ are the learnable parameters of the two FCLs. Through these parameters, weights are generated for each channel. *z* represents the result after squeezing, *δ* represents the ReLU function after the first FCL, and *σ* represents the sigmoid activation function after the second FCL.

Reweighting reweights the original features in the channel dimensions and then sets the output weight of the excitation operation for the original feature by multiplying the channels. The excitation operation is calculated as shown in Formula (4):

$$X_{c}=F_{scale}\left(u_{c},s_{c}\right)=s_{c}*u_{c}$$ (4)

where *X*_*c*_ is the C-th feature map after feature reweighting, *u*_*c*_ is the C-th feature map of the original feature channel, and *s*_*c*_ is the weight of the C-th feature map.

#### Label localization strategy

The use of a fixed label threshold (for example, 0.5) may result in an empty label set for samples whose posterior probabilities are below the fixed threshold. The posterior probability refers to the probability of recorrection after obtaining the "result" information. In this study, it refers to the prediction probability of the test set after the model is optimized. Therefore, in this study, we propose a label localization strategy based on the actual posterior probability. The pretrained classifier is used to output a posterior probability array *P* = {*p*_*ij*_ | *p*_*ij*_ ∈ (0,1), 1 < *j* < *n*} for the test set sample *x*_*i*_(1 < *i* < *m*), and the predicted label set *Y*_*i*_ = {*y* | *y* = 1*or*0} of the sample is obtained through the label localization algorithm.

When annotating a sample, according to the a posteriori probability output by the optimal model for the sample, several maximum probabilities are selected, and the labels represented by these probabilities are used as the sample labels. Note that the number of labels to be selected depends on the dataset and multiple experimental comparisons. The selection process takes the optimal experimental effect into consideration.

### Algorithm flow

First, the pretrained InceptionV3 network is trained on the target dataset based on transfer learning to obtain an optimal model. Next, the optimal model is used to obtain the prediction probability of the test set. Finally, the label localization strategy is employed to select the labels predicted by the model and determine the sample label set. The complete process is as follows:

Input: The processed dataset file
Output: The predicted label set *Y*_*i*_ of the sample
Step 1: Load the weight file (excluding the classifier part) for the InceptionV3 network pretrained on ImageNet.
Step 2: Extract the target dataset features using the pretrained InceptionV3 to obtain an optimal model.
Step 3: Construct the SE module.
Step 4: Define and train the connection classifier.
Step 5: Freeze the bottom-layer convolution operation, and fine-tune and optimize all model parameters.
Step 6: Save the optimal model.
Step 7: Use the optimal model to predict the test set and obtain the predicted probability *P*.
Step 8: Read out the elements in the *i*-th row in *P*, which represent the *i*-th sample in the test sample and are denoted as *p*_*i*_.
Step 9: Select several numbers (the specific number depends on the dataset) with the maximum a posteriori probability in *p*_*i*_ and replace them with 1; the other numbers are replaced with 0.
Step 10: Repeat Steps 8 and 9 until all the sample probabilities have been traversed.
Step 11: The predicted label *Y*_*i*_ of the sample is obtained at this time.

**Fig 3:**
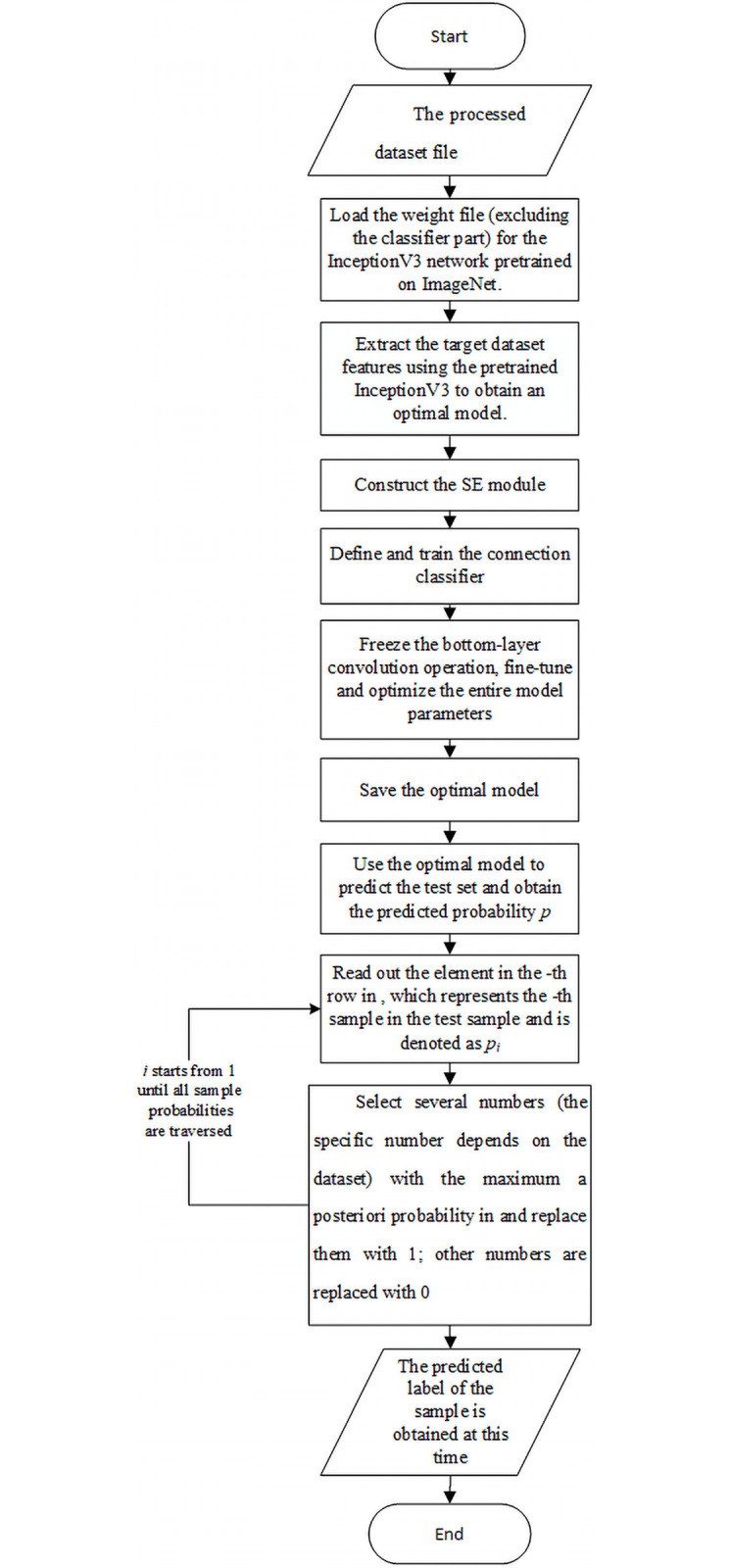
Algorithm flow chart.

The algorithm flow is illustrated in Fig 3.

## Results and discussion

### Experimental data

**Fig 4:**
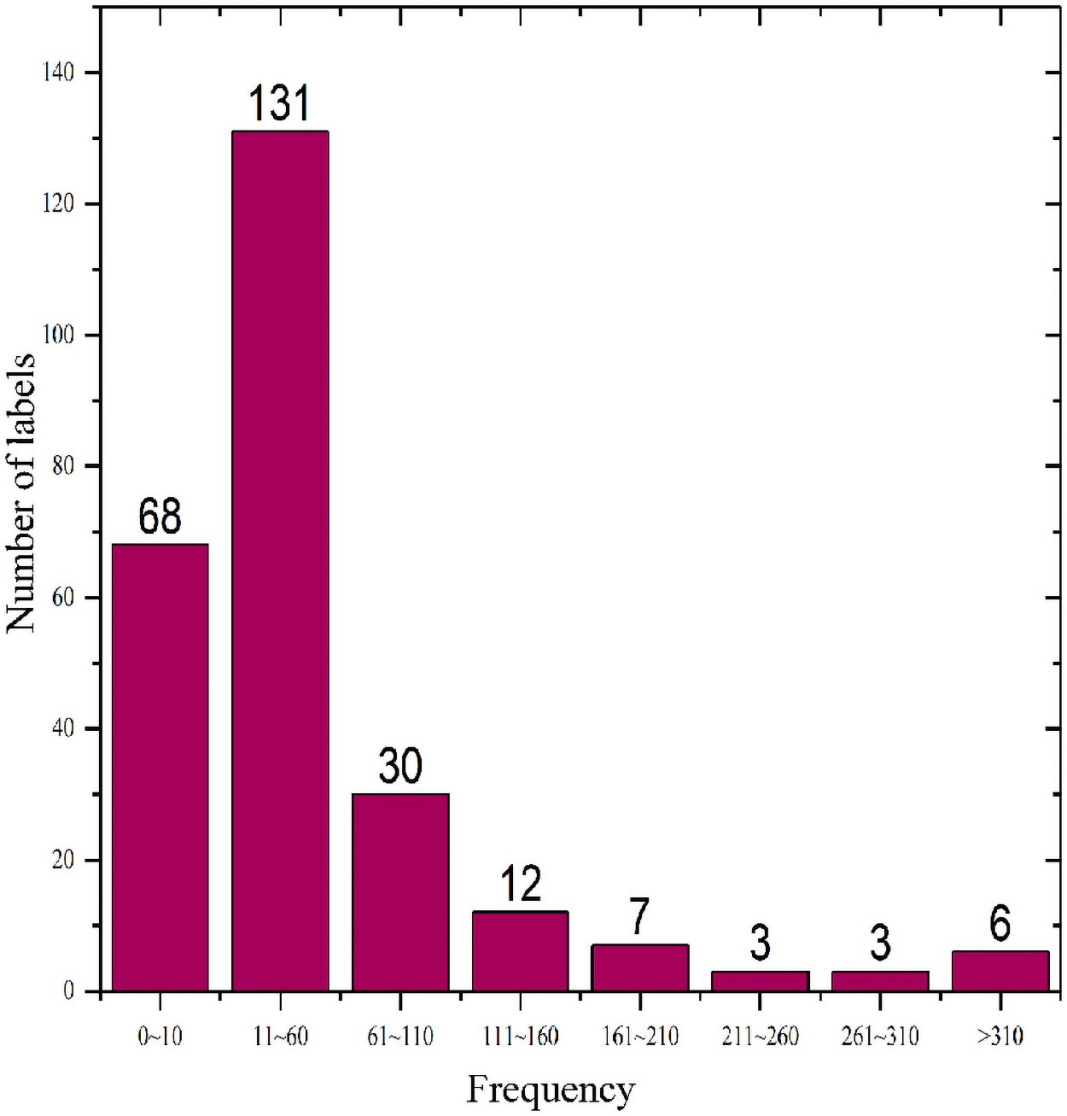
Corel5k dataset label statistics.

To verify the effectiveness of the transfer learning model for automatic image annotation based on the label localization strategy, we use the benchmark Corel5k [26] dataset collected by Corel and the image dataset of natural scenes MIML provided by the Institute of Machine Learning and Data Mining of Nanjing University. The Corel5k dataset has been widely applied for performance comparisons of annotation algorithms. Corel5k contains 4,999 images with sizes of 192×128 or 128×192. Of these, 4,500 are used as the training set and 499 as the test set. When loading the dataset, we uniformly resize all the images to 299×299. There are a total of 260 annotation terms in the dataset; each image is labeled with 1~5 label terms (3.4 labels on average), and each label appears 65.4 times on average. Some of the high-frequency labels in the training set can appear as many as 1,004 times, with a mean of 58.5 times for each label. However, the frequencies of some low-frequency labels are far below the mean. For example, the label for ‘sails’ appears only once in the training set. To intuitively understand the dataset, we compiled the label frequency statistics for the Corel5k training set, as shown in Fig 4.

**Fig 5:**
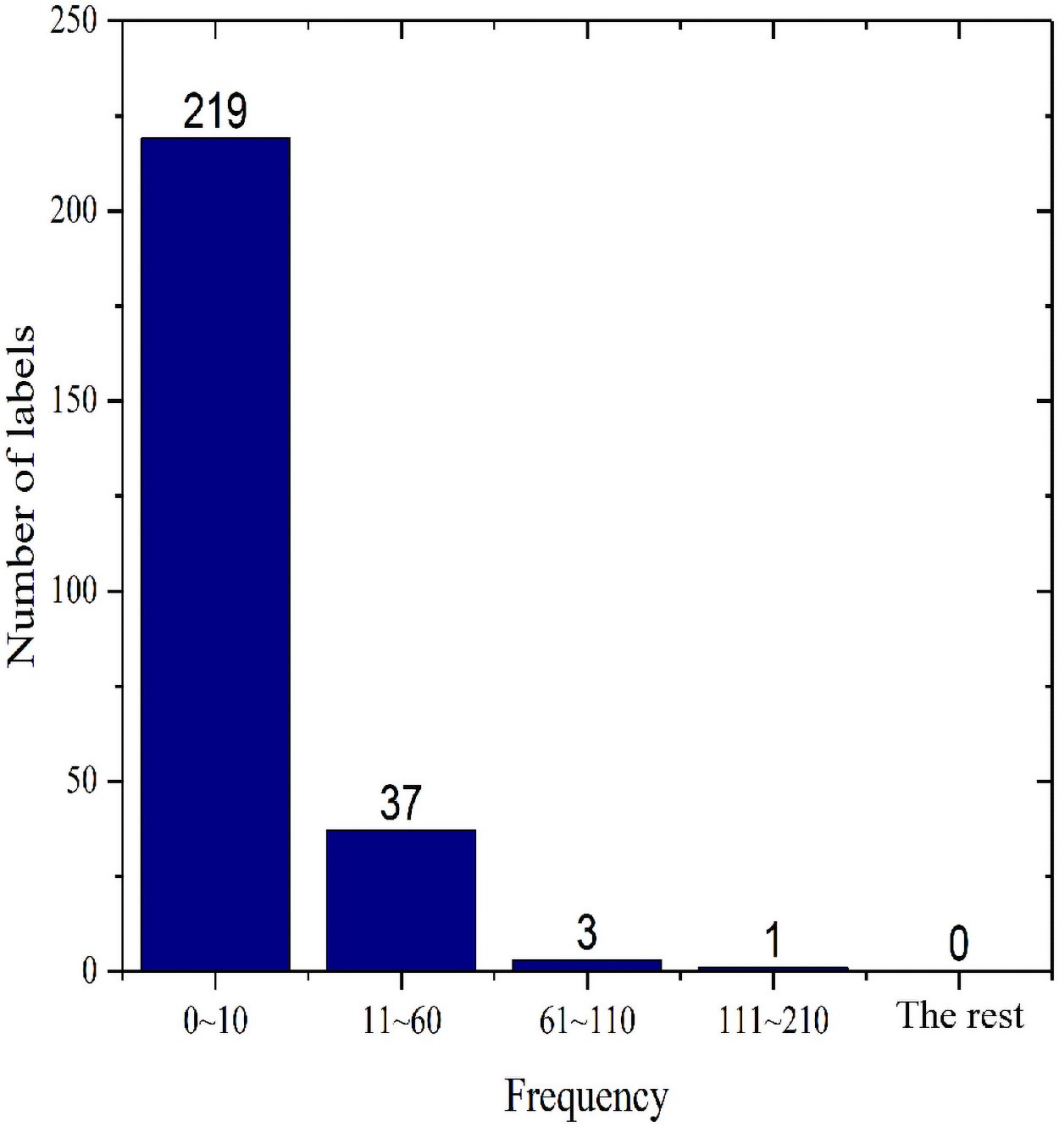
Corel5k test set label frequencies.

Fig 4 clearly shows that the frequency of most labels is well below the mean. Some labels appear fewer than 10 times, and only a few labels appear more often. The model learning effect is good for the high-frequency labels, and the prediction precision can be very high. For some low-frequency labels (0~10), we enhance the images involved to increase their label frequencies to within a range of 11~60, thereby solving the data imbalance issue. Through the statistical analysis, a total of 414 images are associated with 68 low-frequency labels, some of which reappear in the dataset. After removing the repeated images, the number of images that need enhancement is 381, of which the number of images that involve only one label is 3. In this study, those three images are enhanced 12 times, and the remaining 378 images are enhanced 6 times. Finally, the number of images in the Corel5k dataset used for this experiment is 6,922, of which 6,423 are included in the training set and 499 in the test set. The label frequency of the test set is shown in Fig 5.

As shown in Fig 5, among the 260 labels involved in the test set, 219 fall into the low-frequency range of 0~10, 37 fall into the medium frequency range of 11~60, and only 4 labels have a frequency greater than 60.

The MIML dataset contains a total of 2000 images. Among these images, 1600 constitute the training set, and the remaining 400 constitute the test set. For this dataset, five label categories are involved, and the average label number for each image is 1.2.

### Experimental design

We conducted an image annotation simulation experiment based on the Keras deep learning library. To objectively evaluate the effectiveness of the experiment, we selected the precision (*P*), recall (*R*) and *F1* value (F1) as the evaluation indexes, whose calculation formulas are as follows:

$$\text{Average}\;\text{precision}:\;P=\frac{1}{n}\overset{n}{\underset{i=1}{\sum }}\frac{Cor\left(i\right)}{Pre\left(i\right)},$$

$$\text{Average}\;\text{recall}:\;R=\frac{1}{n}\overset{n}{\underset{i=1}{\sum }}\frac{Cor\left(i\right)}{GT\left(i\right)},$$

AverageF1:F1=2PR/(P+R),

where *Cor(i)* is the number of correctly predicted samples for the *i*th category labels, *Pre(i)* is the number of predicted samples for the *i*th category labels, and *GT(i)* is the actual number of samples for the *i*th category labels.

### Analysis of the experimental results

#### Ablation experiment: SE module verification

**Fig 6:**
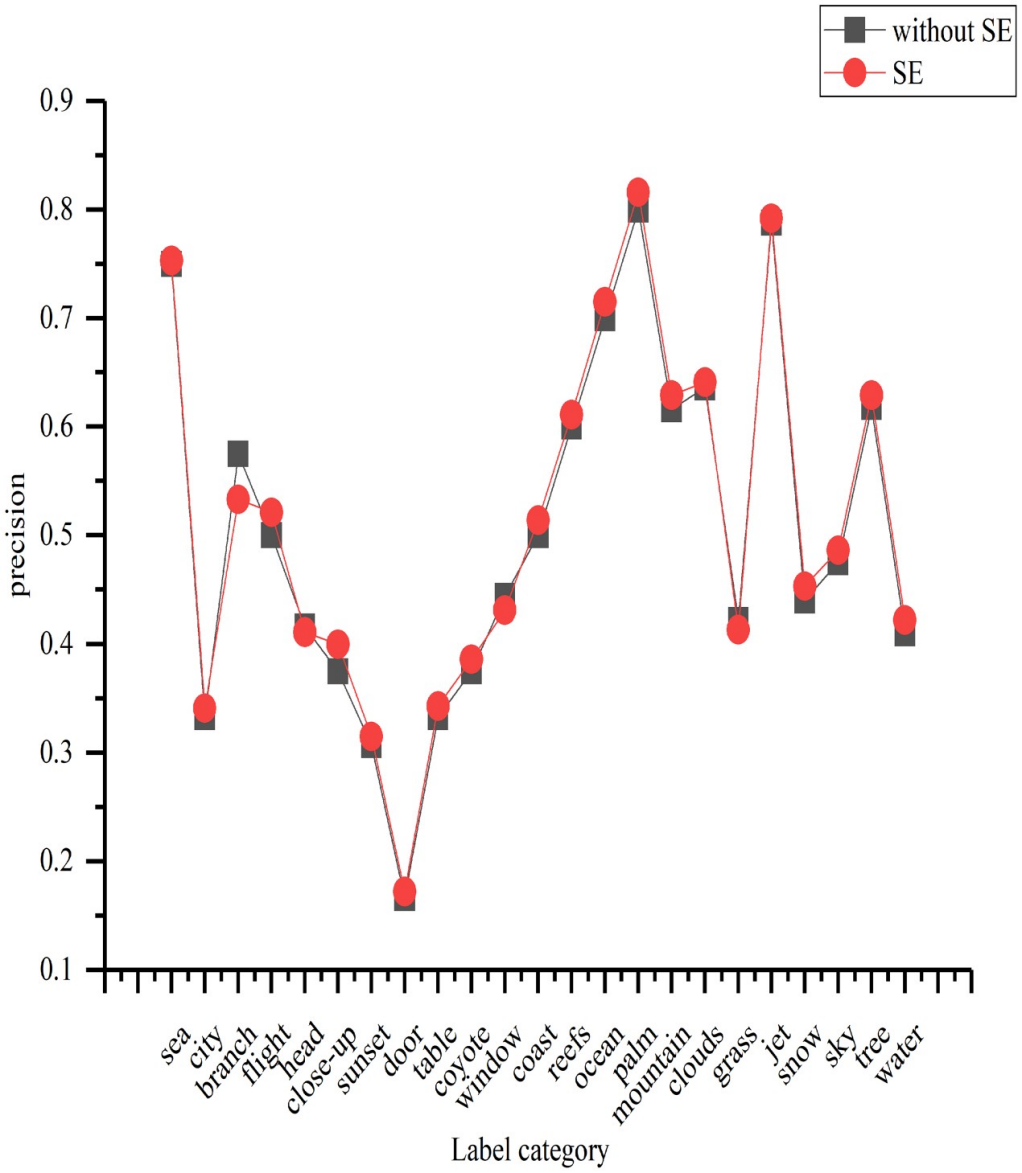
Comparison of the annotation performance with and without the SE module.

To verify that the SE module effectively promotes network performance, we performed an ablation experiment, where we compared the annotation performance of the model with the SE module to that of the same model without the SE module on the Corel5k dataset. The experimental results are shown in Fig 6.

We show only selected labels because there are too many labels in the dataset in this study to display them all. Here, 23 labels are randomly selected according to their proportions to illustrate the effectiveness of the SE module. In Fig 6, the abscissa is the label category; we randomly selected 15 labels from the frequency range of 0–10, 5 labels from the frequency range of 11–60, 2 labels from the frequency range of 61–110, and 1 label from the remaining ranges. The ordinate represents the model precision for each label. As shown in Fig 6, for most labels, the precision of the model with the SE module improves by an average of 1% compared with the model without the SE module. For some labels, such as branches and windows, the precision was not improved; this occurs mainly because the images associated with those labels are not perfectly annotated, and some features that include these labels are not labeled as such. As a result, the label features are treated as useless during channel modeling, which inhibits those features.

#### Label localization strategy verification

To determine the optimal number of label words for the target dataset, we conducted an experiment to compare the output under different numbers of annotation terms (K values). Each K value in Table 1 is selected by the sorting algorithm under the optimal model. By analyzing the experimental results of different K values, we found the optimal K value suitable for the target dataset. Because the mean number of labels for each image in the Corel5k dataset is 3.4, we chose K = 3, 4 and 5 for the experiments in this study given that most previous researchers adopted five labels in their studies. For the MIML dataset, based on full consideration of the average number of labels, 1, 2 and 3 are selected to determine the optimal K value. The results are shown in Table 1.

**Table 1:** Experimental results for different K values.

	K	*P*	*R*	*F1*
Corel5k	3*	0.42	0.38	0.40
	4	0.41	0.43	0.42
	5	0.39	0.50	0.44
MIML	1*	0.826	0.759	0.791
	2	0.803	0.785	0.794
	3	0.786	0.810	0.798

*Indicates the optimal K value for the dataset.

First, it is clear from Table 1 that the experimental results of using the various K values shown in each row are good, indicating that the optimal model obtained in the training is reasonable. Next, as the number of labels (K) increases, *P* decreases while *R* increases. This result occurs primarily because the average number of labels in the Corel5k dataset is 3.4, while that in the MIML dataset is 1.2. When the number of predicted labels is greater than the actual number of labels, that is, the number of predicted samples *Pre(i)* of a certain category of labels increases, it is known according to the precision formula that the corresponding *P* declines. In contrast, as the predicted label categories grow, *R* increases accordingly. According to the different purposes of the experiment, appropriate K values can be chosen. When the experiment needs to recall more label words, K = 5 and K = 3 can be selected as the number of predicted labels when the requirement for precision is not high. The main objective in this study is to improve precision; therefore, we adopted K = 3 and K = 1 as the number of predicted labels.

#### Comparisons with other image annotation methods

When the number of label categories involved in the Corel5k dataset reaches 260, it is not convenient to display them all. Therefore, in this study, we randomly extracted some labels from the low-frequency range of 0~10, some from the medium-frequency range of 11~60, and some from the high-frequency range (above 60). The number of label categories involved in the MIML dataset is small (only five), and the frequency of each category is approximately 500. Therefore, we display all of the annotation outcomes based on the five categories. We make comparisons between our learning transfer model based on the label localization strategy and the traditional image annotation method based on multifeature fusion and semantic similarity in [27] and the Gaussian mixture model that considers cross-modal correlations in [28] (GMM-MB). We also conducted experimental comparisons in terms of precision between the feature fusion model proposed in [29] and the semantic extension model (SEM) proposed in [30], which use a CNN to extract features. The results are shown in Table 2.

**Table 2:** Comparison of annotation precision for single category labels using different algorithms.

Dataset	Frequency	Label category	Annotation precision				
			Literature [27]	GMM-MB [28]	Literature [29]	SEM [30]	CNN-2 L
Corel5k	Low-frequency labels	tulip	0.875	0.896	0.962	0.963	1.000
		sun	0.597	0.632	0.666	0.654	0.684
		sea	0.786	0.775	0.769	0.792	0.800
		palm	0.729	0.730	0.764	0.835	1.000
		fence	0.928	0.922	0.950	0.926	1.000
		runway	0.762	0.769	0.823	0.871	1.000
		flight	0.369	0.413	0.463	0.467	0.500
		head	0.425	0.467	0.521	0.538	0.600
		black	0.551	0.568	0.600	0.619	0.625
		ground	0.913	0.909	0.948	0.949	1.000
		coral	0.612	0.626	0.641	0.653	0.650
		ocean	0.655	0.673	0.687	0.708	0.737
		tiger	0.926	0.941	0.962	0.961	0.950
		fox	0.779	0.776	0.777	0.783	0.800
		arctic	0.906	0.914	0.925	0.990	1.000
		arch	0.527	0.538	0.562	0.605	0.667
		pillar	0.728	0.753	0.768	0.792	0.833
	Medium-frequency labels	mountain	0.564	0.671	0.608	0.612	0.636
		boats	0.589	0.597	0.607	0.643	0.692
		leaf	0.628	0.615	0.644	0.659	0.714
		birds	0.763	0.779	0.778	0.791	0.852
		bridge	0.772	0.792	0.808	0.828	0.875
		plane	0.695	0.716	0.740	0.754	0.789
		bear	0.624	0.710	0.685	0.667	0.719
		polar	0.891	0.926	0.923	0.904	1.000
		flowers	0.652	0.679	0.692	0.735	0.721
		field	0.593	0.605	0.654	0.637	0.680
		plants	0.561	0.629	0.641	0.668	0.643
		pool	0.735	0.731	0.769	0.729	0.786
		cat	0.816	0.847	0.866	0.887	0.900
		ruins	0.682	0.735	0.753	0.815	0.789
		cars	0.694	0.756	0.739	0.742	0.777
		horses	0.847	0.793	0.843	0.896	0.933
	high-frequency labels	sky	0.353	0.351	0.394	0.456	0.475
		tree	0.475	0.472	0.491	0.529	0.618
		people	0.389	0.468	0.524	0.537	0.550
MIML	all labels	desert	0.725	0.769	0.807	0.813	0.826
		mountains	0.766	0.801	0.824	0.816	0.832
		sea	0.713	0.796	0.792	0.835	0.823
		sunset	0.812	0.835	0.843	0.856	0.852
		trees	0.801	0.823	0.822	0.815	0.819

**Fig 7:**
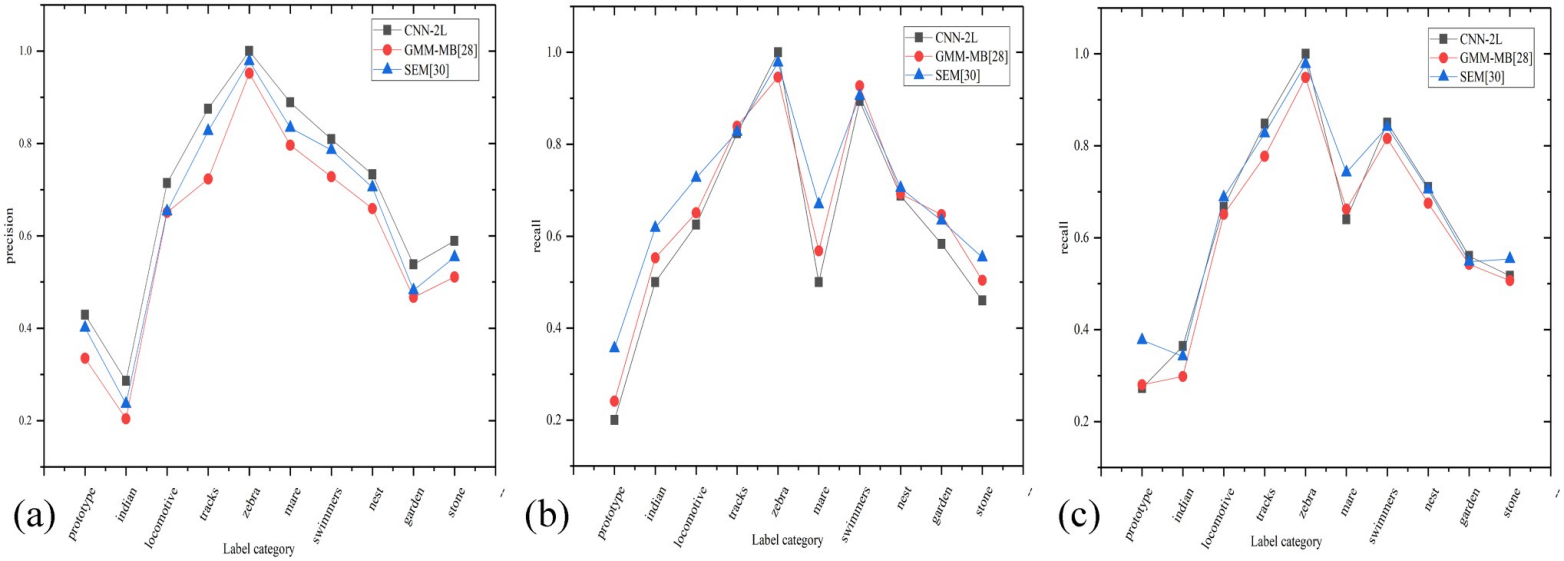
Experimental comparisons of single category indexes under different algorithms.

In addition, we randomly selected some labels from the test results of Corel5k and compared them in an experiment with the traditional method GMM-MB [28] and the deep learning method SEM [30] in terms of the *P*, *R* and *F1* measures, as shown in Fig 7.

As shown in Table 2, for the Corel5k dataset, the CNN-2L model proposed in this study is significantly more accurate than the other methods in terms of annotation precision for single category labels. For categories with distinct features, such as tulip, polar and ground, the annotation precision of all models can reach 100%, while for other labels, such as flight and plane, the annotation precision is low for two reasons: on the one hand, the number of images involved in these categories is limited, which causes imperfect feature learning; on the other hand, the features of these label categories are relatively similar, which introduces some semantic errors. As Fig 7(a) and 7(c) show, compared with the other two methods, the CNN-2L model proposed in this study greatly improves the *P* and *F1* scores for most labels in the dataset for the following reasons: first, by enhancing the number of images involved in low-frequency labels, the model can fully learn the features of each label. Second, the introduced SE module inhibits irrelevant features and enhances the effective features. From Fig 7(b), compared with the other two methods, the *R* of CNN-2L in this study is not improved mainly because CNN-2L adopts a smaller K value during label determination. The experiment shows that although the experimental result obtained by adopting a K value of 3 does not result in an improvement in *R*, it greatly improves the *P* and *F1* scores. Therefore, in contrast to the K = 5 setting adopted by the other models, we adopt K = 3 in this study. For MIML, the number of label categories is small, and the image features are relatively simple. The model can learn the features of each label well. Therefore, the labeling performance of the five labels is excellent.

To verify the effectiveness of the transfer learning model based on the label localization strategy in automatic image annotation, we also compared it with both classical and new models proposed in recent years, such as the multiple-Bernoulli relevance model (MBRM) proposed by Feng et al. [31], the joint equal contribution (JEC) model proposed by Makadia et al. [32], the multifeature fusion and semantic similarity method proposed in [27], the SEM method proposed by Ma et al. [30], the Weight-KNN method proposed in [33], and the adaptive hypergraph learning (AHL) method proposed by Tang et al. [34]. Among these SEMs [30], Weight-KNN [33] and AHL [34] were proposed in the past two years. The experimental results are shown in Table 3.

**Table 3:** Experimental comparison of various automatic image-labeling methods.

Method	Corel5k			MIML		
	P	R	F1	P	R	F1
MBRM [31]	0.24	0.25	0.24	0.53	0.55	0.54
JEC [32]	0.27	0.32	0.29	0.54	0.54	0.54
Literature [27]	0.27	0.33	0.28	0.550	0.56	0.56
Weight-KNN [33]	0.22	0.15	0.18	0.66	0.69	0.67
AHL [34]	0.31	0.38	0.34	0.71	0.73	0.72
SEM [30]	0.37	0.52	0.43	0.77	0.79	0.78
CNN-2L	0.42	0.38	0.44	0.82	0.75	0.78

As Table 3 shows, for the Corel5k dataset, the *P* score of the CNN-2L method proposed in this study is improved by 18% and 15% compared with those of the classical model MBRM [31] and the multifeature fusion and semantic similarity method in [27], respectively. The *R* score is improved by 13% and 6% compared with those of the MBRM [31] and JEC [32] models, respectively. Compared with those of the Weight-KNN [33] and SEM [30] models proposed in the past two years, the *P* score in this study is improved by 20% and 5%, respectively. From the table, it is clear that the *P* score of CNN-2L is higher than those of the other methods, but compared with SEM [30], the *R* of CNN-2L is not improved. This result occurs primarily because the number of predicted labels annotated in this study is relatively small, and some labels with less obvious features are not correctly annotated. However, the *F1* measure of CNN-2L is 1% higher than that of SEM [30]. Over the MIML dataset, the method proposed in this study increases the precision by 29% and 16% compared with the methods used in MBRM [31] and Weight-KNN [33], respectively; it also increases the comprehensive index F1 by 24% compared with JEC [32]. These results indicate the effectiveness of the method proposed in this study.

#### Annotation effect of the proposed model

Table 4 shows the label output for each image by the automatic annotation method. All images involved in these analyses are from the public datasets corel5k and miml-image-data (https://github.com/watersink/Corel5K and http://lamda.nju.edu.cn/files/miml-image-data.rar, respectively). However, due to copyright consideration, we cannot provide the images that are actually analyzed. The method in [27] and the newly proposed AHL [34] are included for comparison with the proposed method.

**Table 4:** Comparison of image annotation methods in various experiments.

Dataset	Ground-truth labels	Label predicted by CNN-2L	Labels predicted by the method in [27]	Labels predicted by AHL [34]
Corel5K	sun water clouds	sun water clouds	sun water birds	sun sky water
	birds		sunset sea	clouds birds
	Plane jet f-16	Plane jet f-16	Plane grass runway f-16 jet	Plane tails jet runway sky
	Sky tree flowers	Sky tree flowers	Sky tree flowers clouds house	Sky tree flowers house clouds
	Tulip			
	Bear polar close-up face	Bear polar snow	Bear face black polar snow	Bear face black close-up snow
MIML	Sea sunset	Sea sunset	Water sea boat people mountains	Sea water boat sunset mountains
	Desert	Desert	Mountains	mountains
			Desert sky grass road	desert sky grass road

Note: Due to copyright consideration, the images actually analyzed in the experiment are not provided.

As shown in Table 4, the method proposed in this study is effective at automatic image annotation, as it predicts most of the annotated words in the ground-truth annotations. Some labels are not predicted successfully because 1) the data volume for those labels is relatively small; thus, the model fails to learn these features well; and 2) the selected K value is relatively small, and therefore, the annotation performance for images containing abundant semantics cannot be perfect. A few labels are incorrectly predicted; although these images do contain the label features, they are not perfectly annotated, and the ground-truth labels do not contain matching annotations. Compared with the other two methods, the method proposed in this study chooses annotations more in accordance with the ground-truth situation for most of the images.

## Conclusions

To address problems such as the insufficiency of available datasets in the multilabel image annotation field and empty label sets caused by a fixed threshold (e.g., 0.5), we propose a transfer learning model based on a label localization strategy for multilabel image annotation. First, using a pretrained CNN model and transfer learning, the features of the target training set are learned well, which solves the issue of insufficient datasets. Moreover, we introduce the SE module into the network architecture, which assigns different weights to the channels during transfer learning. This reweighting process inhibits irrelevant features and enhances useful features. Next, the label localization strategy allocates a fixed number of labels to the image based on the final prediction probabilities of the model, which solves the issue of empty datasets caused when the label probability of an image is smaller than a fixed threshold. The experimental results on the Corel5k dataset show that the proposed CNN-2L model substantially improves the precision, recall and F1 measure results—by 42%, 38% and 44% compared with other methods. The CNN-2L model also improves the precision by 5% compared with the recently proposed SEM [30]. On the MIML dataset, the model proposed in this study achieves a precision of 82%, a recall rate of 75% and an F1 value of 78%; compared with AHL [34], it increases the precision by 11%. These results demonstrate that the proposed method is effective for image annotation applications. The deficiencies of this study are as follows: the overall experiment was divided into two parts: one part involved training an optimal model, and the other involved decision-making regarding the label localization strategy. Overall, these two parts are not well connected. We plan to conduct future studies from two perspectives: 1) improving the loss function and combining the label localization strategy (the second part described in this paper) with the loss function to ensure model integrity; 2) improving the labels for the images in the target datasets by complementing the annotations of unlabeled features to improve the precision in follow-up experiments.
